# Medical residents’ perceptions of their competencies and training needs in health care management: an international comparison

**DOI:** 10.1186/1472-6920-13-25

**Published:** 2013-02-13

**Authors:** Lizanne Berkenbosch, Suzanne Gerdien Schoenmaker, Susannah Ahern, Charlotte Søjnæs, Linda Snell, Albert J J A Scherpbier, Jamiu O Busari

**Affiliations:** 1 School of Health Professions Education (SHE), Faculty of Health, Medicine and Life Sciences, Maastricht University, P.O. Box 616, Maastricht, 6200 MD, the Netherlands; 2 Diaconessenhuis Leiden, Houtlaan 55, Leiden, 2334 CK, the Netherlands; 3 Postgraduate Medical Council of Victoria, St Vincent’s Hospital, PO Box 2900, 41 Victoria Pde, Fitzroy, 3065, Australia; 4 Center for Klinisk Uddannelse, Rigshospitalet, afsnit 5404, Blegdamsvej 9, København, 2100, Denmark; 5 Centre for Medical Education, McGill University, 1110 Pine Ave West, Montreal, QC, H3A1A3, Canada; 6 Maastricht University medical Centre, Postbus 5800, Maastricht, 6202 AZ, the Netherlands; 7 Department of educational research & development, Faculty of Health, Medicine and Life Sciences, Maastricht University, P.O. Box 616, Maastricht, 6200 MD, the Netherlands

**Keywords:** Medical residents, CanMEDS, Managers role, Assessment, International comparison

## Abstract

**Background:**

Previous research has shown that Dutch medical residents feel inadequate in certain management areas: 85% had a need for management training and reported preferences on the format of such training. Our objective was to explore if the perceived deficiencies and needs among Dutch residents were similar to those of their peers in other countries, and if a longer duration of the incorporation of the CanMEDS competency framework into curricula as well as management training had an influence on these perceptions.

**Methods:**

Medical residents from Denmark, Canada and Australia were approached for participation. The questionnaire used to survey the perceptions of Dutch residents was slightly modified, translated into English and sent by email to all international participants.

**Results:**

Response rates were; Denmark 719/2105 (34%), Canada 177/500 (35%) and Australia 194/1213 (16%) respectively. The Danish as well as the Canadian residents rated their negotiating skills poorly. In Australia the residents found their knowledge on how their specialist department was organized to be insufficient, while residents in the Netherlands rated their knowledge on how the healthcare system was organized as inadequate. In all of the countries, more than 70% of the residents reported a perceived need for management training.

**Conclusions:**

A majority of the residents in all countries felt the need for specific training in developing their management competencies. The adoption of the CanMEDS framework alone seems to be insufficient in meeting residents’ perceived educational needs in this area.

## Background

Until now, learning by doing has been the predominant method of training in management competencies for most practicing physicians. In the 1970s some authors proposed that physicians should not only be trained as medical experts, but also as medical managers. Lawson et al (1976) wrote: “Times and methods are changing. Gone are the days when the physician might be paid in kind rather than cash. Gone, too, are the days when one could get by without accounting for profit and loss, when one could be less careful about the source, amount, and use of one’s income. The running of a physician’s office has become a complex business venture” [1]. At the same time, some of the first management training programs were introduced for both staff physicians and trainees [1,2]. These proliferated into the 1990s as more physician management training programs emerged [3-8]. In 1996 the Royal College of Physicians and Surgeons of Canada defined the Managers Role as one of seven key competencies in the CanMEDS framework needed for medical education and practice [9]. A few years later, the Accreditation Council for Graduate Medical Education in the USA identified that Systems-Based Practice was one of the six core competencies for practicing medicine [10].

In 2005 the CanMEDS competencies were officially introduced in the Netherlands as the framework for residency training programs [11], however there is still no formal national curriculum that focuses on the development of management competencies.

In 2009 a literature review of management skill development in postgraduate medical training revealed 40 articles, of which eleven were needs assessments among medical students, residents, fellows or program directors. The general conclusion was that management training was necessary in both the undergraduate and postgraduate training of medical doctors. Twenty-six articles gave a description of existing management courses, which were all different in format and content; all were evaluated positively [12].

In a separate study that investigated the perceived competence and educational needs in health care management of Dutch medical residents, the residents perceived their competencies in certain important medical management areas to be inadequate. More than half of the respondents did not feel confident in their (contract) negotiating skills and lacked sufficient knowledge of how the Dutch healthcare system and the respective specialist practices were financed and organized. About one-third gave neutral ratings on their negotiation and leadership skills and of their knowledge of the legal aspects of healthcare [13]. It was also found that 85% of the residents reported a need for management training [14].

### Current situation of management training in postgraduate medical education

The Royal College of Physicians and Surgeons of Canada (RCPSC) developed the CanMEDS Framework with its seven core competencies in the 1990s; the framework was implemented nationally in 1996 [9]. As residency training in Canada is university-based, any formal Manager Role education is done by the individual programs or the university. The RCPSC instituted a Manager Role ‘Train-the-Trainer’ (TTT) Program to assist residency program directors and university curriculum planners design and implement programs to help residents learn this role. The goals of the TTT addressed curriculum design as applied to the development of Manager Role and developed knowledge in the areas of health care systems, resource management, career development, quality improvement, leadership and change management [15]. The framework was incorporated in Australia in 2006, into the Australian Curriculum Framework for Junior Doctors but provides no formal learning strategies or outlined mechanisms for the formal assessment of the managers’ competency [16]. In 2003, the CanMEDS Framework was adopted in Denmark and in 2005 a formal and mandatory training program was implemented for medical residents covering areas of leadership, collaboration and health care administration to support several of the roles including the manager role [17]. The training program is divided into three courses stretched over nine days. The first course is conducted at the beginning of the specialist training and covers subjects and issues from the physician’s work to illustrate and apply rules and theoretical knowledge about the organization and management at local and regional level. The second and third courses run in the second half of the specialist training. The second course involves issues of health policy, health economics and organizational issues at national, European and international level and the third course covers subjects and issues around leadership, cooperation and organization at local, regional and national level with the doctor as the central actor [18].

So far our research findings on management and leadership training in postgraduate medical education indicate that there is a need for specific training in this area among Dutch medical residents. However, it is unclear whether the perceived deficiencies and needs in management training among Dutch medical residents are similar to those of their peers in other countries. As a result we decided to explore whether the length of incorporation of the CanMEDS framework and the availability or not of management training programs in the postgraduate medical curriculum, influences the perceived management competencies and needs of medical residents in Denmark, Canada and Australia.

## Methods

### Designing the survey

A questionnaire that was designed in 2009 to survey Dutch residents’ management competencies and needs was translated into English and modified to suit the target groups in the different countries [13,14]. For example, we deleted the item on coding and billing in Australia since the junior doctors there do not take part in that administrative process. The final questionnaire consisted of 28 statements about the perceived management competency of junior doctors, which covered four areas of practice management namely: 1. the balance between patient care and personal development, 2. effectively operating within the health care context, 3. allocating healthcare resources appropriately and 4. using information technology to deliver optimal healthcare. The respondents were asked to rank their perceived level of competence on a 5-point Likert-scale, with 1: completely disagreeing, and 5: completely agreeing with a statement. There were seven items which assessed how they perceived their need for management education (training variables). This section not only investigated the need for management training, but also the preferred topics, the preferred method of instruction, when the training should take place and the preferred duration of training. Finally there were six questions about the demographic characteristics of the junior doctors (background variables) i.e. age, gender, years since graduation, current specialty, previous management experience (e.g. in a committee, or previous job) and previous management education (e.g. a management course). We were interested in finding out whether some of these background variables were associated with the junior doctors’ perceptions of their management competencies and management education needs.

### Data collection

Similar to the Dutch study, we made use of an opportunity (convenience) sampling to select our target population, as it was deemed unfeasible to conduct national surveys in these countries due to logistical and time constraints. We emailed the questionnaire to all junior doctors in Denmark that participated in the mandatory courses of pedagogics and communication by the Centre for Clinical Education since 2005. In Australia, hospital based Medical Education Officers forwarded an email to all the junior doctors of eight health services in the state of Victoria. In Canada the questionnaire was forwarded to all residents at McGill University through their respective specialty programs. As the study was a web-based survey (using the Survey Monkey web application) links to the questionnaire were sent to the participants by email. The survey was completed anonymously. The survey was online for six weeks; two reminders (in Canada one) were sent. Prior to starting the survey, we sought for and obtained ethical approval from the McGill Faculty of Medicine Institutional Review Board and the ethical committees of the eight health services in Australia. In Denmark ethical approval is only necessary for biomedical research, for our research project it was therefore not required.

### Data analysis

Analysis was performed using SPSS, version 17. Descriptive statistics were used to present the demographic distribution of the participants. An explorative factor analysis was performed to identify if there were underlying clusters of items (scales) in the questionnaire. Cronbach’s alpha analysis was used to test the reliability of the resulting scales. For the final scales, scale scores were obtained by calculating the mean score of the corresponding items. Whether the residents’ perceptions as expressed by these scale variables were influenced by background variables was investigated using multiple regression analysis. The scale variables formed the dependent variables in the analysis and the background variables were the independent variables. In order to reduce the number of (dummy) variables, the following background variables were selected: Gender, Years since graduation from medical school, Management training and Management experience. Specialty (surgical vs. non-surgical) was not selected as a background variable since previous research indicated that this characteristic had no significant influence on the perceived management competency [13]. Gender was defined 0 = Male, and 1 = Female. Years since graduation was expected to have a skewed distribution and was therefore converted into a logarithmic variable. Management training was defined as 0 = No and 1 = Yes, except for Denmark where no management training was defined as 0, residents who followed the first mandatory course as 1, residents who followed the first and second course as 2, and residents who followed all three courses as 3. Management experience was defined 0 = No and 1 = Yes. The relationship between the perceived need for management training and the background variables mentioned above were investigated by analyzing the corresponding cross tables using Pearson’s Chi-square test to assess the significance of the association. Because years since graduation is a continuous variable, logistic regression was used to assess the effect on the perceived need for management training.

## Results

In Denmark 2105 residents were approached to participate in the survey. Of this total, 719 questionnaires were returned (response rate 34.2%). In Canada 183 out of 500 questionnaires were returned (36.8%) and in Australia 197 out of 1213 surveys (16.2%) were returned. In the Netherlands 177 of 506 residents (35.0%) responded to the survey [13]. The dropout rate (failure to complete the survey) was 8.9% (n = 64) in Denmark, 10.9% (n = 20) in Canada, 9.6% in Australia (n = 19) and 5.1% (n = 9) in the Netherlands. There were no reasons given for not completing the questionnaire. In Denmark 39 respondents were already specialists and excluded from further analysis.

### Residents’ characteristics

The mean age of the Danish residents was 34.2 years (SD 4.3), the mean age of the Canadian, Australian and Dutch residents was 29.2 years (SD 3.9), 27.2 years (SD 3.8) and 30.3 years (SD 3.0) respectively. In Denmark 36.3% of the respondents were male, 50% in Canada 43.3% in Australia and in the Netherlands 33.3%. The average number of years since graduation from medical school was 6.0 years (SD 4.2) for the Danish residents and 3.2 years (SD 2.7), 2.5 years (SD 2.5) and 4.9 years (SD 2.8) for the Canadian, Australian and Dutch residents respectively. Eighty-seven percent of the Danish respondents had participated in management training, in comparison to 13.4% of the Canadian respondents, 14.6% of the Australian respondents and 15.5% of the Dutch respondents. Thirty-eight percent of the Danish residents reported to have some previous experience in a management or leadership function. This percentage was 56.1% for the Canadian residents, 43.8% of the Australian residents and 58.3% of the Dutch residents (Table 1).

**Table 1:** Residents’ characteristics

**Country**		**Netherlands**	**Denmark**	**Australia**	**Canada**
		**(n = 168)**	**(n = 625)**	**(n = 178)**	**(n = 163)**
Gender (Number(%))	Male	56 (33.3)	227 (36.3)	77 (43.3)	81 (49.7)
	Female	112 (66.7)	398 (63.7)	101 (56.7)	82 (50.3)
Age (years) (Mean (SD))		30.3 (3.0)	34.2 (4.3)	27.2 (3.8)	29.2 (3.9)
Specialty (Number(%))	Anesthesiology	15 (8.9)	45 (7.2)	4 (2.2)	6 (3.7)
	Clinical biochemistry	1 (0.6)	10 (1.6)		1 (0.6)
	Clinical genetics	3 (1.8)			
	Clinical microbiology		8 (1.3)		1 (0.6)
	Clinical pharmacology		6 (1.0)		
	Dermatology	6 (3.6)	11 (1.8)	1 (0.6)	2 (1.2)
	ENT medicine	3 (1.8)	10 (1.6)		9 (5.5)
	Emergency department	4 (2.4)		40 (22.5)	7 (4.3)
	General medicine		87 (13.9)	22 (12.4)	34 (20.7)
	Geriatrics		6 (1.0)	6 (3.4)	
	Forensic medicine		3 (0.5)		
	Internal medicine	24 (14.3)	166 (26.6)	16 (9.0)	39 (23.8)
	Intensive care			5 (2.8)	1 (0.6)
	Medical rehabilitation	1 (0.6)		4 (2.2)	
	Neurology (surgery)	7 (4.2)		4 (2.2)	1 (0.6)
	Nuclear medicine		36 (5.8)		
	Obstetrics&Gynecology	31 (18.5)	8 (1.3)	6 (3.4)	6 (3.7)
	Occupational medicine		26 (4.2)	1 (0.6)	
	Ophthalmology	5 (3.0)	3 (0.5)		1 (0.6)
	Orthopedic surgery	5 (3.0)	10 (1.6)	6 (3.4)	7 (4.3)
	Pathology and cytology	5 (3.0)	37 (5.9)		3 (1.8)
	Pediatrics	36 (21.4)	9 (1.4)	5 (2.8)	10 (6.1)
	Psychiatry	2 (1.2)	23 (3.7)	8 (4.5)	11 (6.7)
	Public health		50 (8.0)		1 (0.6)
	Quality&Safety		6 (1.0)	2 (1.1)	
	Radiology	8 (4.8)			6 (3.7)
	Surgery	4 (2.4)	19 (3.0)	32 (18.0)	13 (7.9)
	Urology	3 (1.3)	33 (5.3)	1 (0.6)	3 (1.8)
	Not-stated*	5 (3.0)	13 (2.1)	15 (8.4)	1 (0.6)
Years since graduation (Mean (SD))		4.9 (2.8)	6.0 (4.2)	2.5 (2.5)	3.2 (2.7)
Previous management Training (Number(%))	Yes	26 (15.5)	545 (87.2)	26 (14.6)	22 (13.5)
	No	142 (84.5)	80 (12.8)	152 (85.4)	141 (86.5)
Previous management experience (Number(%))	Yes	98 (58.3)	240 (38.4)	78 (43.8)	91 (55.8)
	No	70 (41.7)	385 (61.6)	100 (56.2)	72 (44.2)

*Not-stated: the question was not answered or the answer was incomprehensible.

### Explorative factor analysis

The questions which did not occur in every survey were removed and for the 26 remaining common competency items the data from the four countries was clustered, using a random 25% (n = 180) sample of the Danish data to create an evenly spread influence from all four countries. An explorative factor analysis (EFA) was performed using 3-5 factors consisting of principal components analysis and oblique rotation. After removal of ambiguous items (loading difference less than 0.1) and low loading items (loading less than 0.4), the EFA was repeated. Using these indications two items were removed, and for the remaining set of 24 items a satisfactory 5-factor solution was obtained (explained variance equal to 65%). Table 2 shows the resulting loadings for this solution (loadings <0.1 not shown), the numbers which are bold are indicating subsets of items associated with each factor (scale). Based on the content of their corresponding items, the five scales were indicated as following: “Management and Leadership”, “Organization and Finance”, “Operational Management”, “Professional Ethics” and “Career Planning (and negotiation skills)” respectively. The reliability (Cronbach’s alpha) of each scale amounted to 0.90, 0.76, 0.84, 0.85 and 0.67 respectively.

**Table 2:** Resulting scales of the explorative factor analysis

	Scales*				
**Items**	**1**	**2**	**3**	**4**	**5**
Time management	**.894**	.103			
Patient care vs. Practice requirements	**.806**				
Patient care vs. Personal life	**.683**				
Leadership skills	**.527**	.118	.246	-.211	
Handling received feedback	**.494**	-.173	.271	-.243	
Managing a ward	**.477**	-.103	.291	-.286	
Using information technology	**.449**	-.191	.333	-.158	
Organisation & finance healthcare system	.137	**.881**	.142		.171
Organisation & finance specialist department		**.866**			
Requirements as specialist		**.630**		-.148	-.209
Allocating resources based on costs	-.188		**.903**		
Allocating resources based on finiteness			**.834**		
Allocating resources based on EBM	.172		**.683**	-.123	
Advocating for patients	.223	-.112	**.525**	-.225	
Responding to mistakes made by others	-.109		-.140	**-.956**	
Responding to mistakes made by myself				**-.865**	
Legal rights and duties	.215			**-.586**	-.121
Proper use of medical records	.272		.135	**-.513**	-.115
Dealing with conflicts	.239	-.138	.181	**-.482**	
Improving quality processes	-.120	.107	.173	**-.457**	-.146
Negotiating personal ambitions				-.104	**-.831**
Create career opportunities	.104		.190		**-.691**
Negotiate working conditions	-.138	.245	-.287		**-.623**
Personal financial management	.284		.158		**-.521**

*Scale 1: Management and Leadership, Scale 2: Organization and Finance, Scale 3: Operational Management, Scale 4: Professional Ethics, Scale 5: Career planning (and negotiation skills).

### Perceived levels of knowledge and skills

In general, residents from Denmark gave their own management competencies a mean score of 3.54 (SD 0.40). The Canadian residents rated their management competencies with an average of 3.35 (SD 0.51), the Australian residents had an average score of 3.45 (SD 0.42) and the Dutch residents 3.39 (SD 0.37). The mean overall scores per scale can be found in Table 3.

**Table 3:** Perceived levels of knowledge and skills

	**Mean (standard deviation)**			
	**Denmark**	**Canada**	**Australia**	**Netherlands**
Mean	3.54 (0.40)	3.35 (0.51)	3.45 (0.42)	3.39 (0.37)
Mean scale 1	3.76 (0.48)	3.74 (0.58)	3.88 (0.45)	3.65 (0.47)
Time management	3.43 (1.00)	3.51 (1.00)	3.67 (0.87)	3.59 (0.89)
Patient care vs. Practice requirements	3.49 (0.84)	3.50 (0.88)	3.90 (0.67)	3.40 (0.90)
Patient care vs. Personal life	3.79 (0.87)	3.64 (0.84)	3.80 (0.78)	3.40 (0.89)
Leadership skills	3.79 (0.76)	3.83 (0.84)	3.67 (0.74)	3.54 (0.70)
Handling received feedback	3.88 (0.65)†	3.84 (0.79)	3.98 (0.61)†	3.92 (0.55)†
Managing a ward	3.88 (0.64)†	3.93 (0.72)†	3.92 (0.55)†	3.78 (0.70)
Using information technology	4.11 (0.69)†	4.01 (0.83)†	4.23 (0.64)†	3.94 (0.87)†
Mean scale 2	3.46 (0.71)	2.93 (0.77)	2.78 (0.78)	2.40 (0.65)
Organisation & finance healthcare system	3.55 (0.84)	3.23 (0.98)	2.86 (0.97)*	2.55 (0.84)
Organisation & finance specialist dep.	3.42 (0.89)	2.66 (0.97)*	2.59 (0.92)*	2.11 (0.77)*
Requirements as specialist	3.39 (0.93)	2.92 (0.96)	2.88 (0.98)	2.53 (0.81)*
Mean scale 3	3.55 (0.51)	3.74 (0.64)	3.40 (0.62)	3.75 (0.49)
Allocating resources based on costs	3.28 (0.86)	3.47 (0.88)	3.16 (0.86)	3.42 (0.86)
Allocating resources based on finiteness	3.39 (0.71)	3.71 (0.82)	3.46 (0.86)	3.65 (0.70)
Allocating resources based on EBM	3.76 (0.76)	3.81 (0.68)	3.44 (0.82)	3.90 (0.58)
Advocating for patients	3.76 (0.76)	3.95 (0.81)†	3.54 (0.80)	4.03 (0.60)†
Mean scale 4	3.55 (0.53)	3.28 (0.66)	3.52 (0.58)	3.51 (0.48)
Responding to mistakes made by others	3.38 (0.83)	3.07 (0.90)	3.29 (0.89)	3.33 (0.80)
Responding to mistakes made by myself	3.69 (0.77)	3.38 (0.83)	3.59 (0.83)	3.53 (0.75)
Legal rights and duties	3.80 (0.73)	3.29 (0.96)	3.66 (0.69)	3.49 (0.75)
Proper use of medical records	3.82 (0.76)	3.43 (0.91)	3.76 (0.81)	3.62 (0.74)
Dealing with conflicts	3.33 (0.85)	3.56 (0.88)	3.54 (0.73)	3.72 (0.64)
Improving quality processes	3.25 (0.94)*	2.94 (0.97)	3.29 (0.96)	3.38 (0.87)
Mean scale 5	3.27 (0.67)	2.74 (0.79)	3.18 (0.65)	3.16 (0.63)
Negotiating personal ambitions	3.10 (0.98)*	2.58 (1.00)*	3.11 (0.93)	3.38 (0.86)
Create career opportunities	3.71 (0.82)	2.89 (1.02)	3.30 (0.82)	3.42 (0.85)
Negotiate working conditions	2.51 (0.97)*	2.23 (0.91)*	2.75 (0.93)*	2.45 (0.87)*
Personal financial management	3.75 (0.92)	3.26 (1.09)	3.54 (0.89)	3.36 (0.94)

*Three lowest scores per country, † Three highest scores per country.

The three lowest rated items from the Danish residents were “negotiating working conditions” 2.51 (SD 0.97), “negotiating personal ambitions” 3.10 (SD 0.98) and “improving quality processes” 3.25 (SD 0.94). For the Canadian residents the three lowest rated items were “negotiating working conditions” 2.13 (SD 0.91), “negotiating personal ambitions” 2.58 (SD 1.00) and “organization specialist department” 2.66 (SD 0.97). The three lowest rated items for the Australian residents were “organization of a specialist department” 2.59 (SD 0.92), “negotiating working conditions” 2.75 (SD 0.93) and “organization of the healthcare system” 2.86 (SD 0.97). The Dutch residents three lowest rated items were “organization of the healthcare system” 2.11 (SD 0.77), “negotiating working conditions” 2.45 (SD 0.87) and “requirements as a specialist” 2.53 (SD 0.81).

The three items the Danish residents rated highest were “using information technology” 4.11 (SD 0.69), “handling received feedback” 3.88 (SD 0.65) and “managing a ward” 3.89 (SD 0.64). The three highest rated items among the Canadian residents were “using information technology” 4.01 (SD 0.83), “standing up for patients” 3.95 (SD 0.81) and “managing a ward” 3.93 (SD 0.72). The three highest rated items for the Australian residents were “using information technology” 4.23 (SD 0.65), “handling received feedback” 3.98 (SD 0.61) and “managing a ward” 3.92 (SD 0.55). The three highest rated items for the Dutch residents were “standing up for patients” 4.03 (SD 0.60), “using information technology” 3.94 (SD 0.87) and “handling received feedback“ 3.92 (SD 0.55), as can be seen in Table 3.

### Multiple regression analysis per country

#### Denmark

As is shown in Table 4 there were three variables, which had a significant influence on the overall perceived management competencies of the Danish residents. The first variable was “gender” (b = -.108, p = .001), the male residents gave themselves on average significantly higher scores in comparison to their female counterparts. The second variable was “previous management experience” (b = .181, p = .000), where the residents with previous management experience gave themselves significant higher ratings than their colleagues without such experience. The third factor was “previous management course” (b = .082, p = .001), where the residents with more training scored on average increasingly higher (mean of no training 3.40 (SD 0.38); mean of one training 3.55 (SD 0.37); mean of two training courses 3.57 (SD 0.38) and the residents who participated in all three training courses had a mean of 3.83 (SD 0.27)). These three variables were also of significant influence in the factor “Organization and Finance” (gender b = -.148, p = .016, experience b = .281, p = .000 and training b = .121, p = .010). In the factor “Management and Leadership” the variable gender had a significant influence (b = -.139, p = .001), as did the variable training (b = .070, p = .027). In the factor “Operational Management” the variables experience and training were of significant influence on the average ratings of the residents (b = .133, p = 0.002 and b = .126, p = .000 respectively). In the factor “Professional Ethics” gender, experience and years since graduation had a significant influence (b = -.097, p = .033, b = .210, p = .000 and b = .187, p = .012 respectively). And in the last factor “Career Planning” the variables gender and previous management experience were of significant influence (b = -.224, p = .000 and b = .297, p = .000 respectively).

**Table 4:** Results of the multiple regression analysis

**Dependent variable**	**Gender**			**Management experience**			**Years since graduation**			**Management course**		
	**b**	**beta**	**p**	**b**	**beta**	**p**	**b**	**beta**	**p**	**b**	**beta**	**p**
Denmark												
Overall	.108	.135	.001	.181	.229	.000			NS	.082	.160	.001
Management and Leadership	.139	.142	.001			NS			NS	.111	.027	.027
Organization and Finance	.148	.099	.016	.281	.190	.000			NS	.121	.047	.010
Operational Management			NS	.133	.125	.003			NS	.126	.034	.000
Professional Ethics	.097	.088	.033	.210	.193	.000	.187	.123	.012			NS
Career planning	.224	.161	.000	.297	.215	.000			NS			NS
Canada												
Overall			NS	.292	.290	.000	.201	.166	.037			NS
Management and Leadership			NS	.235	.205	.014			NS			NS
Organization and Finance			NS	.392	.255	.002			NS	.435	.192	.018
Operational Management			NS			NS			NS			NS
Professional Ethics			NS	.226	.173	.037	.310	.197	.015			NS
Career planning			NS	.506	.319	.000	.487	.255	.001			NS
Australia												
Overall			NS	.178	.216	.007			NS			NS
Management and Leadership			NS			NS	.244	.174	.023			NS
Organization and Finance			NS			NS			NS			NS
Operational Management			NS	.203	.164	.041			NS			NS
Professional Ethics			NS	.188	.163	.046			NS			NS
Career planning	.270	.203	.008			NS			NS			NS
Netherlands												
Overall			NS	.172	.238	.003			NS			NS
Management and Leadership			NS			NS			NS			NS
Organization and Finance			NS	.292	.237	.003			NS			NS
Operational Management			NS			NS			NS			NS
Professional Ethics			NS	.200	.208	.011			NS			NS
Career planning			NS			NS			NS			NS

b = regression coefficient.

beta = standard regression coefficient.

p = significant.

NS = non-significant.

#### Canada

The two variables which had a significant positive influence on the overall mean scores of the Canadian residents were “previous management experience” (b = .292, p = 0.000) and “years since graduation” (b = .201, p = 0.037). These two variables were also of influence in the factors “Professional Ethics” (experience b = .226, p = 0.037 and graduation b = .310, p = 0.015) and in “career planning” (experience b = .506, p = 0.000 and graduation b = .487 p = 0.001). In the factor “Management and leadership” only previous management experience had a significant influence (b = -.235, p = 0.014). In the factor “Organization and Finance” previous management experience had a positive significant influence (b.392, p = 0.002), but a previous management course had a negative significant influence (b = -.435, p = 0.018).

#### Australia

The only variable which had an influence on the average overall score was “previous experience” (b = .178, p = 0.07); residents who had previous management experience gave themselves on average higher scores. This effect was also seen in the factors “operational management” (b = .203, p = 0.041) and professional ethics (b = .188, p = 0.046). In the factor “management and leadership”, residents who had more years since graduation gave on average lower ratings than their colleagues with fewer years since graduation (b = -.244, p = 0.023). In the factor “career planning” female residents gave themselves lower scores on average than their male counterparts (b = -.270, p = 0.008).

#### Netherlands

In the Netherlands only the variable previous experience had an influence on the average ratings of the residents (b = .172, p = 0.003), the residents who had this experience rated themselves on average higher. This effect was also found in the factors Organization and Finance (b = 0.292, p = 0.003) and Professional ethics” (b = .200, p = 0.011) [13].

### Needs assessment

More than fifty percent of the residents from all four countries felt that there wasn’t sufficient attention being given to management training in their current residency program. The vast majority of residents in all four countries had a need for management training (Denmark 84.7%, Canada 83.5%, Australia 76.8% and the Netherlands 85.3%). When we analyzed the different subcategories in Denmark we saw that 84.6% of Danish residents who had not received any training yet, had a need for management training. The percentages of residents who perceived a need for additional management training after a first training course was 87.0%, after two training courses 81.2% and after all three training courses 78.9% respectively.

The top three preferred topics for a management training program differed between countries except for “negotiation skills” which was in the top three of preferred topics in all four countries. Two topics that were least preferred in all countries included knowledge of “computer systems” and “electronic databases”. The favorite method of instruction was the same for Canada (70.6%), Australia (68.5%) and the Netherlands (84.7%), namely a workshop. The residents from Denmark preferred a case based method (63.6%). The majority of all residents chose “physicians” or “extramural experts (e.g. a lawyer)” as preferred instructors for such programs. The hospital manager was chosen least frequently in all four countries. The preferred length of each training differed between Denmark, Canada and Australia from one hour to a half day. The preferred frequency of the sessions among those respondents varied between once every month or every half-year. In the Netherlands however, the preferred length of training and training frequency was investigated using open questions. The respondents’ preferred duration of the training ranged from 1 to 400 hours, with a mean of 17 hours. The length of the program that they preferred most was one that was spread over days (n = 42 times) or years (n = 43 times). A training period spread over months (n = 19 times) or weeks (n = 2 times) was not a preferred choice [14]. The majority of all residents chose the postgraduate period as being the most suitable period for management training rather than during medical school or after specialization (Denmark 87.3%, Canada 85.3%, Australia 75.7%, the Netherlands 95.3%) (Table 5).

**Table 5:** Residents’ needs (Times chosen (%))

**Country**		**Denmark**	**Canada**	**Australia**	**Netherlands**
		**(n = 632)**	**(n = 170)**	**(n = 181)**	**(n = 170)**
Sufficient attention*	Totally disagree	26 (4.1)	27 (15.9)	21 (11.6)	17 (10.0)
	Disagree	335 (53.0)	66 (38.8)	73 (40.3)	69 (40.6)
	Neutral	174 (27.5)	41 (24.1)	54 (29.8)	43 (25.3)
	Agree	79 (12.5)	33 (19.4)	28 (15.5)	39 (22.9)
	Totally agree	18 (2.8)	3 (1.8)	5 (2.8)	2 (1.2)
Needs training	Yes	535 (84.7)	142 (83.5)	139 (76.8)	145 (85.3)
	No	97 (15.3)	28 (16.5)	42 (23.2)	25 (14.7)
Top three preferred topics	Leadership skills	413 (65.3)			
	Negotiation skills	358 (56.6)	107 (62.9)	113 (62.4)	119 (70.0)
	Cost-effectiveness	284 (44.9)		92 (50.8)	
	Career		128 (75.3)	109 (60.2)	
	Financial planning		111 (65.3)	92 (50.8)	
	Specialist department				108 (63.5)
	Healthcare system				97 (57.1)
Top three least preferred topics	Computer systems	91 (14.4)	33 (19.4)	21 (11.6)	13 (7.6)
	Private practice	134 (21.2)			
	Electronic database	154 (24.4)	35 (20.6)	19 (10.5)	21 (12.4)
	Communication skills		30 (17.6)	34 (18.8)	
	Cost-effectiveness				18 (10.6)
Top three method of instruction	Case based	402 (63.6)			56 (32.9)
	Interactive	355 (56.2)	85 (50.0)	107 (59.1)	64 (37.6)
	Workshop	350 (55.4)	120 (70.6)	124 (68.5)	144 (84.7)
	Lecture		83 (48.8)	102 (56.4)	
Preferred Instructor	Physician	534 (84.5)	148 (87.1)	153 (84.5)	140 (82.4)
	Expert**	404 (63.9)	115 (67.6)	108 (59.7)	140 (82.4)
	Manager	184 (29.1)	43 (25.3)	53 (29.3)	36 (21.2)
Top two preferred length each teaching	One hour			86 (47.5)	†
	Two hours		54 (31.8)	59 (32.6)	
	One day	322 (50.9)			
	Half day	129 (20.4)	68 (40.0)		
Top two teaching sessions every.	Month	220 (34.8)	56 (32.9)	72 (39.8)	†
	Half year	200 (31.6)	68 (40.0)	39 (21.5)	
Training should occur during.	Medical school	201 (31.8)	51 (30.0)	92 (50.8)	42 (24.7)
	Postgraduate training	552 (87.3)	145 (85.3)	137 (75.7)	162 (95.3)
	As a specialist	341 (54.0)	30 (17.6)	43 (23.8)	32 (18.8)

*Sufficient attention is given to management training in my residency program.

**Expert: extramural expert, e.g. a lawyer.

† This was an open question in the Dutch questionnaire, for the answer see section “needs assessment”.

### Influences on the perceived need for management training per country

#### Denmark

Gender was a significant influence on the perceived need for management training. Significantly more female residents had a need for management training than their male counterparts (p = .004). Previous management training (p = .291) and previous management experience (p = .206) were not a significant influence on the need for management training.

Also, the years since graduation was not a significant influence on the perceived need for management training (p = .131).

#### Canada

Gender, previous management training and previous management experience were not a significant influence on the residents need for management training (p > .05). The years since graduation did have a significant influence, the more time had passed the less need there was for management training (b = -2.52, p = .000).

#### Australia

Female residents had on average a significant greater need for management training than their male counterparts (p = .015). Previous management training was not a significant influence, residents who did not have previous management experience had a significant greater need for management training than their colleagues who did have such experience (p = .007). Logistic regression showed that the more years since graduation passed the more need residents felt for a management training (b = 1.86, p = .009).

#### The Netherlands

Gender was not an influence on the perceived need for management training (p = .283). Neither was previous management training (p = .263), previous management experience (p = .256) or the years since graduation (p = .798) [14].

## Discussion

In this study we investigated whether the perceived deficiencies and needs among Dutch residents were similar to those of their peers in other countries and if a longer incorporation of the CanMEDS framework and management training had an influence on the residents’ perceptions of their management competency.

We used a questionnaire that measured the perceived management competencies of residents from Denmark, Canada, the Netherlands and Australia and found out that on the average, residents from Canada scored lowest in their perceived competencies. In addition, the Canadian residents had lesser number of years of clinical experience post graduation than their Danish and Dutch counterparts and multiple regression analysis showed that this factor significantly influenced the average perception of their management competencies. This finding was remarkable however, since the CanMEDS framework emerged from Canada and had been implemented in their postgraduate training program about 10 years prior to its implementation in the other countries included in our survey. The Canadian respondents scored especially low, in comparison to the residents from the other countries, on the items in the factors Career Planning and Professional Ethics. Our assumption is that this may be due to the fact that the general guidelines for postgraduate training programs set up by the RCPSC are subject to the interpretation of the different program directors and how they subsequently translate these guidelines into specific educational activities. It is possible for example, that residents do not receive any training in these areas, because some program directors may feel that residents will learn this on the job, or that they may get more training in their senior years. Why the Dutch residents gave higher ratings than their colleagues in Canada is not completely clear, since they too do not receive any mandatory management training during medical school as well as during the postgraduate training. An explanation for the higher ratings from the Australian junior doctors could be that in some universities a number of hours are spent on topics such as health services management and health economics [personal communication: S. Ahern, Medical Director of Postgraduate Medical Council of Victoria, Australia].

The residents from Denmark gave themselves on average the highest scores, which could be due to the mandatory management training, which all residents in Denmark receive. We saw in our results that the more training they received, the higher their average score was (mean no training 3.40 (SD 0.38), mean one training 3.55 (SD 0.37), mean two training courses 3.57 (SD 0.38) mean three training courses 3.83 (SD 0.27)). Moreover the medical students in Denmark also receive a three-week course on Medical Sociology where topics such as “knowledge of how the healthcare system is organized and financed”, “management technologies”, “quality development” and “health policies” are being addressed [19].

The item “negotiating working conditions” was in the top three of lowest perceived competencies in all four countries. “Knowledge of how their respective specialist department is organized and financed” was in the top three of lowest scored items in Canada, Australia and the Netherlands while the other items in the respective top three category differed per country. These differences can, for example, be attributed to the structure of the health care systems the residents were working in, or to the education they received in (medical) school. These differences should be accounted for when developing and designing a management training in the different countries.

The same can be said about the residents preferred topics for a future management course. The topics differed per country except for “negotiation skills” which was in the top three of preferred topics in all of the countries. Since the majority of the residents from all four countries rated their negotiation skills lowly and chose it as a preferred topic for a management course, it suggests that this topic is a subject residents want to know more about and that it is a need which is felt in all participating countries.

Most importantly, the majority of the residents from all four countries felt a need for (more) management training. In Denmark, despite the extra attention that was given to the managers’ role during the postgraduate training, the average need for additional management training remained high. The different subcategories showed that the percentage of Danish residents who had not received any training yet and had a need for management training was 84.6%. The percentages of residents who had an additional management training need and received one, two or all three training courses was 87.0%, 81.2% and 78.9% respectively. An explanation for the increase in percentage after the first training could be that their interest in the subject is being raised after their first management course and that awareness arises of their knowledge gap in this area.

The favorite method of instruction was the same for Canada, Australia and the Netherlands, namely a workshop. The residents from Denmark preferred a case based method. The majority of all residents chose “physicians” as the preferred instructor for a management-training program. The preferred length of each training differed between Denmark, Canada and Australia from one hour to a half day. The preferred frequency of sessions among the respondents from those three countries was once every month or every half year.

We acknowledge that there is little room left for additional content in most postgraduate medical curricula and that including yet another element, namely medical management, into these busy programs may be difficult. However we think that since the managers role has been identified as a key competency in the four participating countries, the training programs in those countries need to design courses to develop this competency. Also, the extent to which these skills would need to be taught may differ for instance by the resident’s number of years in training or their personal interest. Nevertheless, our research showed that the majority of the residents felt a need for a basic understanding of medical management.

As we mentioned earlier in our methods section, there were constraints e.g. logistical and time that influenced our approach and choice for convenience sampling. As this could have resulted in our survey population being biased, we need to interpret our findings with caution. For example, the response rate was not as high as we had hoped for in all four countries and particularly low in Australia, despite the two reminders we sent. We assume that the low response rates could have been due to the increasing amount of emails and questionnaires that residents receive, which may have resulted in a lack of time and interest in the residents to respond. We know this to be especially true for the Australian residents, as they regularly receive surveys from their own health services. Furthermore, our use of convenience sampling in the Netherlands, Australia and Canada may have resulted in a selection of samples that were not fully representative of the situation among all the residents at a national level in the respective countries. In other words, our findings in the district of Victoria may not necessarily be representative of a national view or of the view of residents in other districts. However, we do expect some degree of generalizability in the results since all countries have similar frameworks (CanMEDS) in their national health services, which have been used to develop their respective professional training programs. Finally, caution should be exercised when interpreting these results as we measured the perceived management competencies of the residents in our survey, and it is possible that the scores could have been different if objective assessment methods had been used.

## Conclusion

In conclusion, the incorporation of the CanMEDS framework alone does not seem to be enough to improve the perceived management competencies among medical residents. The majority of the residents from all four countries had a need for management training, despite the duration of the incorporation of the CanMEDS framework and whether or not management training was provided in their programmes. Furthermore, receiving formal management training seemed to positively influence perceived management competencies. While we acknowledge that any recommendation for additional management training within the medical curriculum would undoubtedly be at the cost of the attention given to development of other professional competencies, there is sufficient evidence to suggest that the introduction of formal management training in postgraduate training programmes should be made mandatory, and that these should be adjusted to the specific needs of the residents in every country.

## Competing interests

The authors declare that they have no competing interests.

## Authors' contributions

LB has made substantial contributions to conception and design, acquisition of data, analysis and interpretation of data. LB also has been involved in drafting the manuscript and gave final approval of the version to be published. SS has made substantial contributions to acquisition of data, analysis and interpretation of data. SS also revised the manuscript critically for important intellectual content and gave final approval of the version to be published. SA has made substantial contributions to acquisition of data. SA also revised the manuscript critically for important intellectual content and gave final approval of the version to be published. CS has made substantial contributions to acquisition of data. SA also revised the manuscript critically for important intellectual content and gave final approval of the version to be published. LS has made substantial contributions to acquisition of data. SA also revised the manuscript critically for important intellectual content and gave final approval of the version to be published. AS has made substantial contributions to conception and design. AS also revised the manuscript critically for important intellectual content and gave final approval of the version to be published. JB has made substantial contributions to conception and design and the acquisition of data. JB also has been involved in drafting the manuscript, revised the manuscript critically for important intellectual content and gave final approval of the version to be published.

## Pre-publication history

The pre-publication history for this paper can be accessed here:

http://www.biomedcentral.com/1472-6920/13/25/prepub

## References

[B1] Lawson JG McConnell JW Teaching practice management in a family practice residency J Med Educ 1976 51 858 860 972380 10.1097/00001888-197610000-00012

[B2] Aluise JJ The physician as manager. “What” and “HOW” OF PRACTICE MANAGEMENT EDUCATION J Fam Pract 1977 4 305 311 65441

[B3] Piatt JP Bartley DL Jacobson AD Rimsza ME Practice management training for pediatric residents Am J Dis Child 1991 145 299 301 2003479 10.1001/archpedi.1991.02160030067023

[B4] Sims KL Darcy TP A leadership-management training curriculum for pathology residents Am J Clin Pathol 1997 108 90 95 9208984

[B5] Brooks JP Suggestions for management training of residents Physician Exec 1996 22 26 28 10155972

[B6] Farnsworth JR Weiss RL A mentor-based laboratory management elective for residents Am J Clin Pathol 1999 111 156 160 9930135 10.1093/ajcp/111.2.156

[B7] Cordes DH Rea DF Rea J Vuturo A A program of management training for residents Acad Med 1989 64 45 46 10.1097/00001888-198901000-00017 2914065

[B8] Winkelman JW Brugnara C Management training for pathology residents. II. Experience with a focused curriculum Am J Clin Pathol 1994 101 564 568 8178761 10.1093/ajcp/101.5.564

[B9] CanMEDS: better standards, better physicians, better care http://www.royalcollege.ca/portal/page/portal/rc/canmeds

[B10] MOC Competencies and Criteria. http://www.abms.org/Maintenance_of_Certification/MOC_competencies.aspx

[B11] Modernisering medische vervolgopleidingen: nieuw kaderbesluit CCMS (Dutch) http://knmg.artsennet.nl/Opleiding-en-Registratie/Algemene-informatie/Nieuws/O-R-Nieuwsartikel/Modernisering-medische-vervolgopleidingen-nieuw-kaderbesluit-CCMS.htm

[B12] Busari JO Berkenbosch L Brouns JW Physicians as managers of health care delivery and the implications for postgraduate medical training: a literature review Teach Learn Med 2011 23 186 196 10.1080/10401334.2011.561760 21516608

[B13] Berkenbosch L Brouns JW Heyligers I Busari JO How Dutch medical residents perceive their competency as manager in the revised postgraduate medical curriculum Postgrad Med J 2011 87 680 687 10.1136/pgmj.2010.110254 21693572

[B14] Brouns JW Berkenbosch L Ploemen-Suijker FD Heyligers I Busari JO Medical residents perceptions of the need for management education in the postgraduate curriculum: a preliminary study Int J Med Educ 2010 1 76 82

[B15] Train-the-Trainer workshop resource binders http://www.royalcollege.ca/portal/page/portal/rc/canmeds/resources/workshops

[B16] Australian Curriculum Framework for Junior Doctors (ACF) http://www.cpmec.org.au/Page/australian-curriculum-framework-for-junior-doctors-acf-menu 10.5694/j.1326-5377.2007.tb00959.x 17407415

[B17] Specialist Commission MoH The future specialist (Danish) 2000 Copenhagen: Ministry of Health 167 167

[B18] Vejledning om generelle kurser i speciallægeuddannelsen (Danish) http:/www.retsinformation.dk/Forms/R0710.aspx?id=136870

[B19] Studieordning for kandidatuddannelsen i medicin (Danish) http://medicin.ku.dk/om_uddannelsen/studieordninger/

